# Efficacy of Continuous Positive Airway Pressure on Testosterone in Men with Obstructive Sleep Apnea: A Meta-Analysis

**DOI:** 10.1371/journal.pone.0115033

**Published:** 2014-12-11

**Authors:** Xiao-Bin Zhang, Xing-Tang Jiang, Yan-Ping Du, Ya-Ting Yuan, Bo Chen

**Affiliations:** Department of Respiratory Medicine, Zhongshan Hospital, Xiamen University, Xiamen, China; Weill Cornell Medical College Qatar, Qatar

## Abstract

**Objective:**

To evaluate the efficacy of continuous positive airway pressure (CPAP) on serum testosterone in men with obstructive sleep apnea (OSA).

**Methods:**

Two reviewers independently searched PubMed, Cochrane library, Embase and Web of Science before June 2014. Information on characteristics of subjects, study design, pre- and post-CPAP treatment of serum total testosterone, free testosterone and sexual hormone blinding protein (SHBG) was extracted for analysis.

**Results:**

A total of 7 studies with 9 cohorts that included 232 men were pooled into meta-analysis. There was no change of total testosterone levels before and after CPAP treatment in OSA men (standardized mean difference (SMD) = −0.14, 95%*CI*: −0.63 to 0.34, z = 0.59, *p* = 0.558), even subdivided by CPAP therapeutic duration (>3 months). Meanwhile, no significant differences in free testosterone and SHBG were detected after CPAP treatment (SMD =  0.16, 95%*CI*: −0.09 to 0.40, z = 1.25, *p* = 0.211 and SMD = −0.58, 95%*CI*: −1.30 to 0.14, z = 1.59, *p* = 0.112, respectively).

**Conclusion:**

CPAP has no influence on testosterone levels in men with OSA, further large-scale, well-design interventional investigation is needed.

## Introduction

Obstructive sleep apnea (OSA) is a common disorder, affecting approximately 4% of the male and 2% of the female middle-aged adults [1]. The characteristics of OSA are intermittent hypoxia and sleep fragmentation. The pathogenesis of OSA is the interaction of the following factors: anatomical abnormality of upper airway and ventilator control instability [2].

Accumulating evidence showed that OSA is correlated with endocrinal dysfunction, disorder of hypothalamic-pituitary-gonadal axis [3]–[8], Sexual dysfunction, particularly erectile dysfunction (ED), is common in OSA men [9], and ED can been ameliorated via continuous positive airway pressure (CPAP), the standard treatment modality of OSA [10], [11]. Testosterone is one of sexual hormones and terminal part of hypothalamic-pituitary-gonadal axis. Robust evidence confirmed that low testosterone is tightly associated with sexual dysfunction [12], and testosterone administration can improve sexual dysfunction in men [13], [14]. Several studies found that low serum testosterone levels were observed in patients with OSA [15]–[17]. Some investigations indicated that testosterone administration, however, aggravates the severity of OSA [18], [19]. Previous data recommended that CPAP can improve sexual dysfunction and endocrinal disorder [10], [11], [20], [21], however, whether serum testosterone levels may be ameliorated or not by CPAP is unclear.

The aim of the present meta-analysis was to quantitatively evaluate the impact of CPAP on testosterone in men with OSA.

## Methods

### Search strategy

The electronic databases PubMed, Cochrane Library, Embase and Web of Science were searched by two reviewers (XB Zhang and B Chen). The combination of the following terms was used: ‘sleep apnea’, ‘continuous positive airway pressure’, ‘testosterone’. We searched articles published from their inception to June, 15th, 2014. The reference lists of the included studies were also hand-searched.

### Selection criteria

Studies were included if they met the following criteria: 1) participants enrolled in the studies were male, if study reported the data of both gender, only the male data was extracted. 2)OSA was diagnosed with overnight polysomnography; 3) the studies must report serum testosterone levels as primary outcome or secondary outcome before and after application of CPAP; 4) studies must provide sufficient data for meta-analysis. Excluded criteria were as following: 1)studies which did not satisfy the criteria would be excluded; 2)non-English article; 3)studies presented as correspondence, review, case report; 4)studies whose essential data were presented as median and interquartile range; 5)unpublished data from conference. If the required data of studies was ambiguous, the corresponding author was contacted, after two no response attempt, the studies were also ruled out. Any disagreement between the two reviewers(XB Zhang and B Chen) were resolved by discussing with a third reviewer(XT Jiang).

### Data extraction

Two reviewers (XB Zhang and YT Yuan) independently evaluated the included studies. The following variables were retrieved: first author, publication year, country of the study, participant characteristics, study design, CPAP using duration, serum testosterone levels before and after CPAP treatment. If study population included both men and women, only male data were extracted for analysis. Jadad score [22] was used to assess the quality of randomized controlled trial (RCT).

### Statistical analysis

Statistical analysis was performed using Stata Version 12.0 (Stata Corporation, College Station, Texas, USA) and Review manager 5.2. Standardized mean difference (SMD) and 95%confidence interval (*CI*) were calculated for presenting continuous outcomes. I-square (*I^2^*) was used to present statistical heterogeneity. Random effects model was performed to combine effect size if *I^2^*>50%, otherwise, fixed effects model was conducted. Sensitivity analysis was conducted to investigate the influence of a single study on overall efficacy of CPAP. Publication bias was presented using funnel plot and tested by ‘Begg test’ and ‘Egger test’. A *p* value less than 0.05 was adopted as statistical significance.

## Results

### Searching results

**Figure 1:**
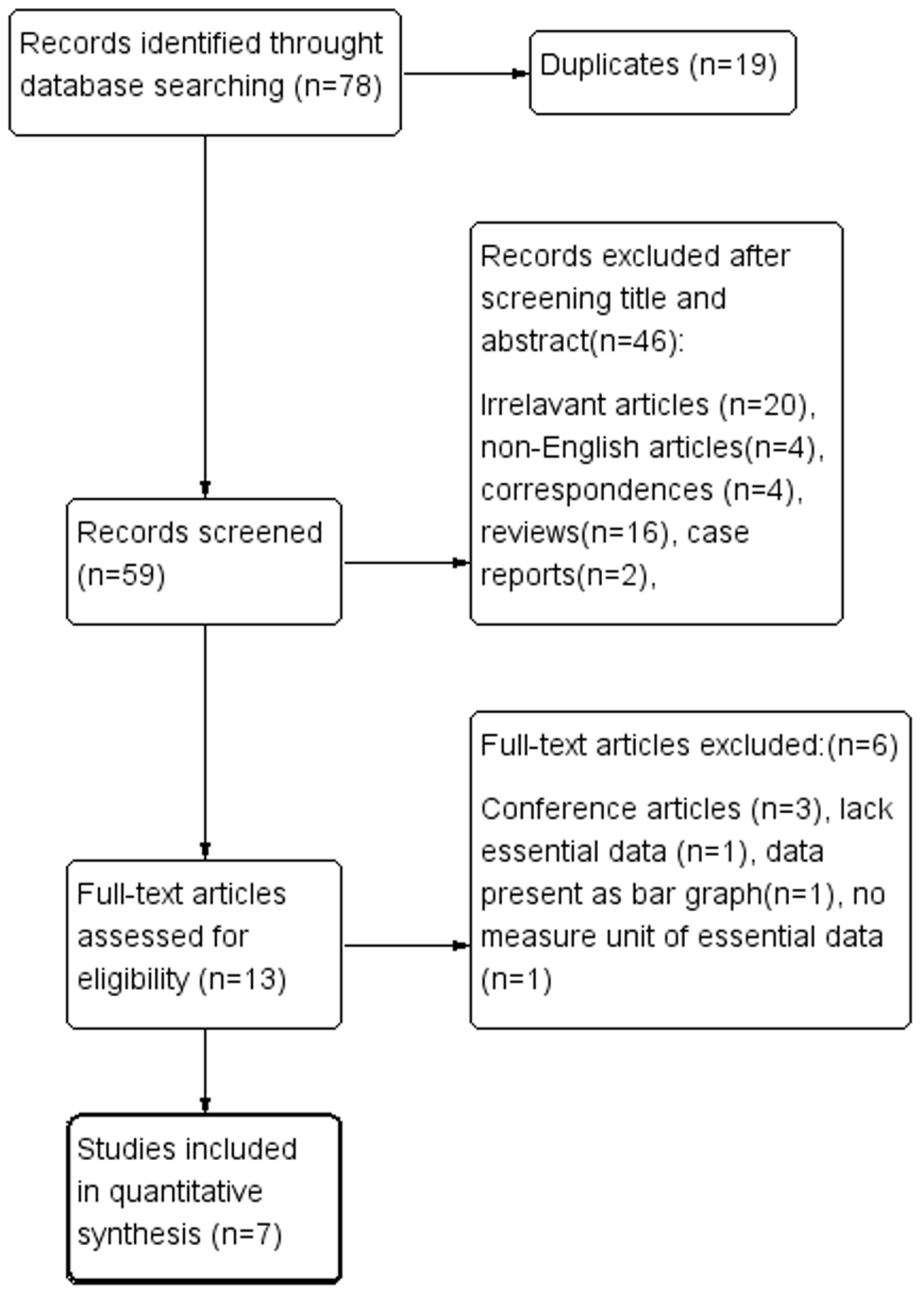
Study flow diagram.

A total of 59 studies were retrieved to screen after searching duplication. Forty-six studies were exclude after browsing the titles and abstracts. The remaining 13 studies were selected for full-text review. Of the 13 studies, 6 were further exclude for following reasons: 3 were conference articles [23]–[25], 1 lacked essential data [26], the data of one study present as bar graph [27] and one study had no measure unit of essential data [28]. A total of 7 studies [7], [9], [20], [21], [29]–[31] (9 cohorts) pooled for final meta-analysis. Two of them were RCT [9], [21], 5 were observational study. The study flow diagram is outlined in Fig. 1.

### Characteristics of the included studies

The 7 studies (9 cohorts) with 232 men were entered into the meta-analysis. One study reported the data from men and women, only the data of men was extracted [30]. One study reported results separately for 1 month group and 6 months group [30], another study reported results separately for 1 month and 3 months [31]. All 7 studies with 9 cohorts presented total testosterone levels before and after CPAP treatment, 4 studies with 5 cohorts reported free testosterone levels [7], [9], [29], [31], and sex hormone binding protein (SHBG) was reported in 4 studies with 5 cohorts [7], [21], [29], [31]. The characteristics of the included studies and the relevant data are outlined in Table 1. Two RCT studies are summarized separately in Table 2.

**Table 1:** Characteristics of include studies (7 studies, 9 cohorts).

Author	Year	Age (mean ±SD)	BMI(mean ±SD)	AHI(baseline)	Study Design	Duration(months)	CPAP daily use(h)	Sample (baseline/after treatment)	Total testosterone (mean±SD, nomol/l)		Free testosterone (mean±SD, nomol/l)		SHBG (mean±SD, nmol/l)	
									baseline	After treatment	baseline	After treatment	baseline	After treatment
Grunstein [29]	1989	NA	NA	NA	Observational	3	NA	43/43	14.4±1.2	17.0±1.1	0.512±0.047	0.509±0.035	19.8±1.9	22.7±2.0
Bratel [7]	1999	51.3±3.7&	32.0±1.6&	43.3±4.7	Observational	7	NA	11/11	17.7±1.8	16.7±2.1	0.5±0.07(n = 3)	0.43±0.04(n = 7)	13.7±1.9(n = 3)	27.1±6.5(n = 7)
Luboshitzky [20]	2003	49.5±5.2	31.7±4.7	56.0±22.4*	Observational	9	5.2±2.4	5/5	2.9±6.7	2.6±11.3	NA	NA	NA	NA
Meston [21]	2003	NA	NA	NA	RCT^∧^	1	5.4±1.6	52/52	13.5±5.8	13.2±4.7	NA	NA	26.8±10.8	29.9±15.1
Hoekema [9]	2007	51±9	32.0±3.0	46.7 (10–64.6)#	RCT^∧^	2.5@	6.3±1.3	27/27	16.0±4.7	16.2±5.2	0.37±0.11	0.39±0.13	NA	NA
Celec 1 month [30]	2014	56.2±9.4	33.7±4.3	54.5±16.6*	Observational	1	NA	67/67	20.7±7.45	21.0±8.4	NA	NA	NA	NA
Celec 6 months [30]	2014	56.2±9.4	33.7±4.3	54.5±16.6*	Observational	6	NA	67/67	20.7±7.45	20.1±7.32	NA	NA	NA	NA
Knap 1 month [31]	2014	65.4±9.6	32.5±5.2	NA	Observational	1	5.5±1.8	27/27	12.7±4.5	11.5±4.0	0.26±0.07	0.23±0.06	32.3±11.6	31.1±13.2
Knap 3 months [31]	2014	65.4±9.6	32.5±5.2	NA	Observational	3	5.1±1.9	27/27	12.7±4.5	11.9±3.5	0.26±0.07	0.25±0.05	32.3±11.6	31.1±12.5

SHGB:sex hormone binding protein; RCT: randomized controlled trial. *:Presented as respiratory disturbance index(RDI), #: Presented as median (interquartile range), &:n = 16, ^∧^: Jadad score of both RCT>3, @:the CPAP duration in the original article was 76 days.

**Table 2:** Characteristics of two randomized controlled trials.

Author	Year	Study design	Treatment group				Control group					Jadad score
			Samples	Total testosterone(baseline, nmol/l)	Total testosterone(after treatment,nmol/l)	*p* value	Type	Samples	Total testosterone(baseline, nmol/l)	Total testosterone(after treatment, nmol/l)	*p* value	
Meston [21]	2003	Parallel	52	13.5±5.8	13.2±4.7	0.94	Sham CPAP	49	14.4±5.0	12.9±4.4	0.004	3
Hoekema [9]	2007	Parallel	27	16.0±4.7	16.2±5.2	>0.05	Oral appliance	20	19.1±6.9	17.9±5.8	>0.05	3

### Meta-analysis on total testosterone, free testosterone and SHBG

**Figure 2:**
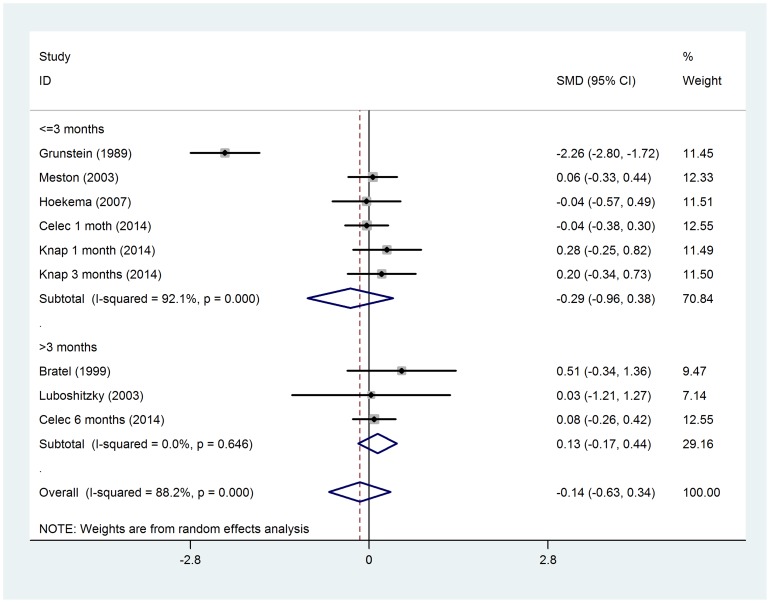
Forest plot for the change in total testosterone before and after CPAP treatment.

**Figure 3:**
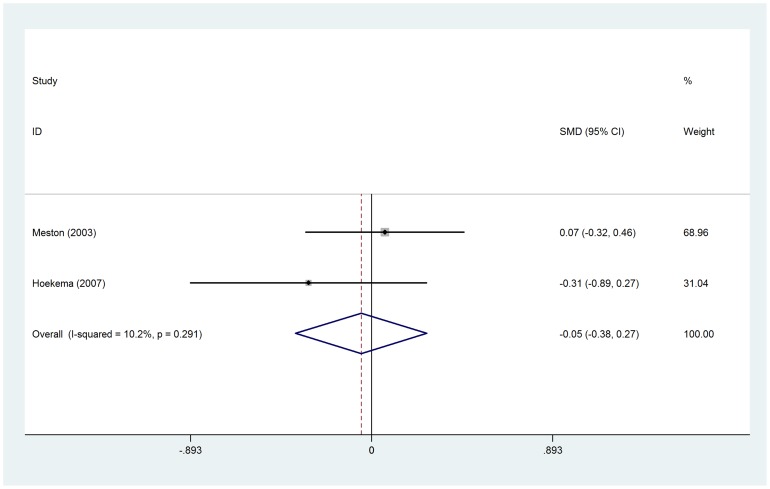
Forest plot for the change in total testosterone between CPAP treatment group and control group in two RCTs.

**Figure 4:**
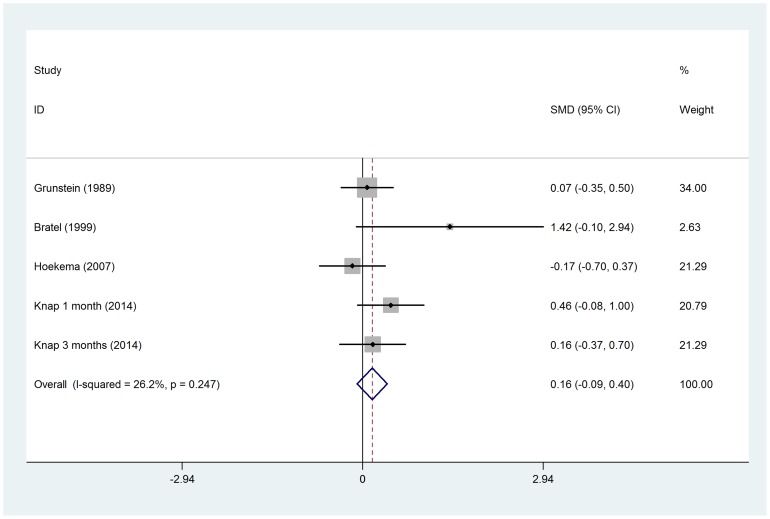
Forest plot for the change in free testosterone before and after CPAP treatment.

**Figure 5:**
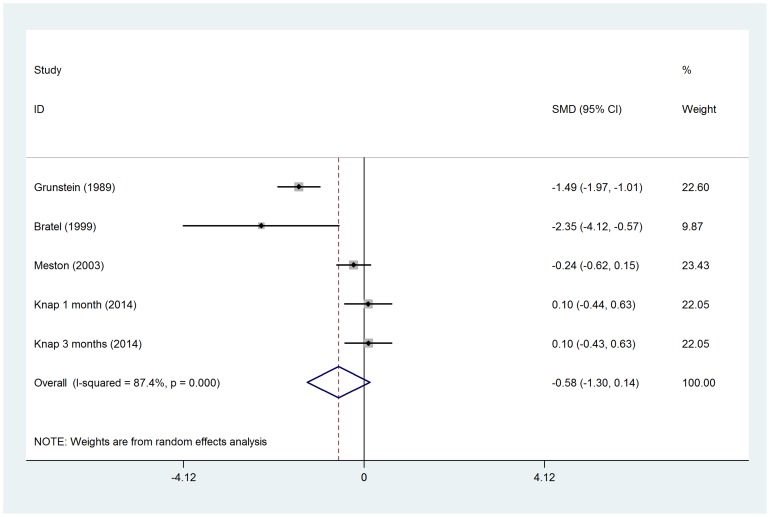
Forest plot for the change in SHBG before and after CPAP treatment.

No significant difference in total testosterone was observed before and after CPAP treatment (SMD = −0.14, 95%*CI*: −0.63 to 0.34, z = 0.59, *p* = 0.558), even after subgroup analysis by CPAP therapy duration (Fig. 2). Two RCTs were separately analyzed, when compared with control groups (sham CPAP or oral appliance), testosterone levels was no changed in CPAP group (SMD = −0.05, 95%*CI*: −0.38 to 0.27, z = 0.31, *p* = 0.757) (Fig. 3). The changes in free testosterone and SHBG had also not got statistical significances between pre- and post-CPAP treatment (SMD =  0.16, 95%*CI*: −0.09 to 0.40, z = 1.25, *p* = 0.211 and SMD = −0.58, 95%*CI*: −1.30 to 0.14, z = 1.59, *p* = 0.112, respectively) (Figs. 4 and 5). Even after omitting the study of Grunstein's [29], the SMD changed to 0.082, sensitivity analysis had not yet reached statistical significance (95%*CI*:−0.083 to 0.247) (Table 3).

**Table 3:** Sensitivity analysis of 7 including studies (9 cohorts).

Study omitted	Estimate	(95%Confidential interval)
Grunstein [29]	0.082	(−0.083 to 0.247)
Bratel [7]	−0.214	(−0.729 to 0.302)
Luboshitzky [20]	−0.158	(−0.670 to 0.353)
Meston [21]	−0.171	(−0.739 to 0.398)
Hoekema [9]	−0.157	(−0.703 to 0.389)
Celec 1 month[30]	−0.157	(−0.742 to 0.428)
Celec 6 months [30]	−0.174	(−0.753to 0.405)
Knap 1 month [31]	−0.199	(−0.736 to 0.337)
Knap 3 months [31]	−0.188	(−0.729 to 0.352)
Combined	−0.145	(−0.630 to 0.358)

### Publication bias

**Figure 6:**
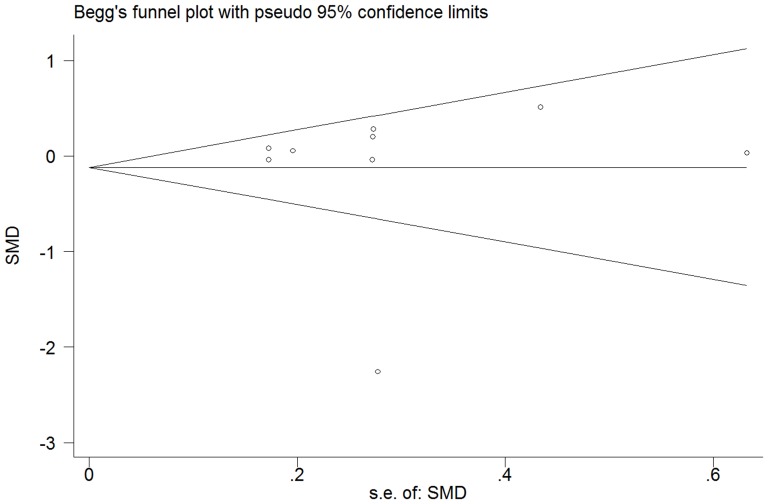
Publication bias.

Fig. 6 shows that publication bias seem to exist, however, both Begg and Egger tests proved that there is no strong statistical evidence for publication bias in the present meta-analysis (*p* = 0.917 and 0.823, respectively).

## Discussion

The present study with 7 articles and 9 cohorts quantitatively evaluated the efficacy of CPAP on testosterone in men with OSA. Subsequently, subgroup and sensitivity analysis were conducted to further analysis. The result of present study indicated that CPAP has not impact on testosterone, irrespective of CPAP duration and study design.

To our best knowledge, present study was the first meta-analysis addressing the effect of CPAP on testosterone in OSA men. Our meta-analysis has several strengths. Firstly, pooling of the interesting data from all eligible studies yielded more precise and reliable conclusions than the data from individual study. Secondly, Two RCTs with relatively high Jadad scores were enrolled in our meta-analysis, it strengthened our conclusion. Thirdly, there was no strong statistical evidence for publication bias in our meta-analysis. Fourthly, we performed sensitivity analysis and subgroup analysis subdivided by CPAP therapeutic duration, both the results did not show a statistical significance.

Several limitations of our study have to be mentioned. Firstly, Most of the included studies were before and after treatment study, only two RCTs, pre-and post-treatment data rather than treatment and control groups data were drawn. It may, to some extent, weaken the reliability of our conclusions. Secondly, the small sample size restrict the extrapolation of our conclusion, additional large-scale, well-designed and long-term RCT investigation is required. Thirdly, since data was extracted from men in each individual study, the conclusion may be unreasonable to extrapolate to women. Fourthly, only papers published in English were enrolled, it may cause potential publication bias.

OSA can impair hypothalamic-pituitary-adrenal/gonadal axis, causing endocrinal and sexual dysfunction, and these dysfunction can be ameliorated by CPAP therapy [9], [20], [32]. However, the exact role of intermittent hypoxia and sleep fragmentation in the correlation between OSA, endocrinal dysfunction and sexual dysfunction still needs further study to elucidate. Testosterone, considered as one of sexual hormones, is negatively correlated with the severity of OSA and sexual dysfunction in men [8], [12], [17]. Although men with OSA have low testosterone levels, some investigations have found that testosterone administration aggravates OSA [18], [33], the causality between testosterone and OSA is still unclear. CPAP is the first choice for OSA treatment. As mentioned above, previous studies have confirmed the efficacy of CPAP on endocrinal disordering and sexual dysfunction in OSA patients [9], [20], [32], however, whether serum testosterone can be normalized or not by CPAP in OSA patients is controversial. In the present meta-analysis, excepting Grunstein and colleague [29] early in 1989 suggested that CPAP can improve testosterone in 43 OSA men, the remaining 6 studies (including 2 RCTs) with 8 cohorts elucidated that there is no influence of CPAP on serum total testosterone, free testosterone and SHBG in men with OSA. Additional further subgroup analysis showed that the results did not change when subdivided by CPAP duration (< =  3 months and>3 months). Meanwhile, sensitivity analysis was conducted to omit each including study, the pooled SMD was not yet changed in the meta-influence analysis.

In conclusion, the present meta-analysis demonstrated that CPAP treatment does not improve testosterone levels in OSA men, irrespective of CPAP therapeutic duration and study design.

## Supporting Information

S1 Figure **PRISMA Flow Diagram.**
(DOC)

S1 PRISMA Checklist 10.1371/journal.pone.0115033.s002
(DOC)
