# Supplementary material for: Efficacy of Continuous Positive Airway Pressure on Testosterone in Men with Obstructive Sleep Apnea: A Meta-Analysis
Source: PLoS One. 2014 Dec 11;9(12):e115033. doi: 10.1371/journal.pone.0115033 (PMC4263732; doi:10.1371/journal.pone.0115033)
Supplement: S1 Figure — PRISMA Flow Diagram. (DOC) [file pone.0115033.s001.doc]

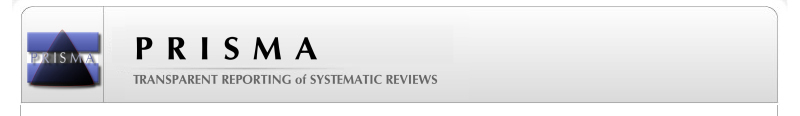
**PRISMA 2009 Flow Diagram**

**Screening**

**Included**

**Eligibility**

**Identification**

Records identified through database searching
(n =78 )

Additional records identified through other sources
(n = 0 )

Records after duplicates removed
(n = 59 )

Records screened
(n = 59 )

Records excluded
(n =46 )

Full-text articles assessed for eligibility
(n =13 )

Full-text articles excluded, with reasons
(n =6 )

Studies included in qualitative synthesis
(n = 7 )

Studies included in quantitative synthesis (meta-analysis)
(n = 7)
